# Precision-targeted nanodelivery systems for intra-articular treatment of knee osteoarthritis: recent breakthroughs and translational challenges

**DOI:** 10.3389/fbioe.2026.1777056

**Published:** 2026-05-18

**Authors:** Yongli Zhao, Yaxiong Gao, Wenhua Zhao, Fadong Li, WeiBing Hou, Tiansheng Bu

**Affiliations:** 1 Affiliated Hospital of Gansu University of Chinese Medicine, Lanzhou, China; 2 School of Food and Biological Engineering, Hefei University of Technology, Hefei, Anhui, China; 3 Gansu University of Chinese Medicine, Lanzhou, Gansu, China; 4 Gansu Health Vocational College, Lanzhou, Gansu, China; 5 The First People’s Hospital of Baiyin City, Baiyin, Gansu, China

**Keywords:** cartilage penetration, intra-articular nanodelivery, knee osteoarthritis, mesenchymal stem cell–derived exosomes, precision-targeted nanoparticles, stimuli-responsive release, theranostic nanoplatforms

## Abstract

Knee osteoarthritis (KOA) is a progressive whole-joint disorder characterized by cartilage degeneration, synovial inflammation, subchondral bone remodeling, and pathological alterations of the intra-articular (IA) microenvironment. Conventional IA therapies—including corticosteroids, hyaluronic acid, and emerging biologics—provide only transient relief due to rapid synovial clearance, poor cartilage penetration, and limited targeting specificity. Recent advances in nanotechnology have enabled the development of precision-targeted IA nanodelivery systems capable of overcoming anatomical, biomechanical, and biochemical barriers inherent to the osteoarthritic joint. This review summarizes recent breakthroughs in the rational engineering of nanocarriers, including optimization of physicochemical parameters, cartilage- and cell-specific targeting ligands, microenvironment-responsive release mechanisms, lubricating and mechanically adaptive nanomaterials, and integrated theranostic platforms. Progress in preclinical rodent and large-animal models is evaluated, alongside emerging translational evidence and early clinical experience. Remaining challenges—including long-term biocompatibility, pharmacokinetic heterogeneity across OA phenotypes, scalable manufacturing, and regulatory classification—are critically discussed. We highlight future directions centered on personalized nanotherapy, artificial intelligence-assisted carrier design, human-relevant joint organoids, and phenotype-stratified clinical trials. Collectively, precision-targeted IA nanodelivery represents a promising frontier toward clinically viable disease-modifying osteoarthritis drugs (DMOADs) and may ultimately shift KOA treatment from symptomatic management to true disease modification.

## 1 Introduction

Osteoarthritis (OA) is the most common form of arthritis and a leading cause of disability worldwide, particularly affecting the knee joint due to its weight-bearing nature (Steinmetz et al., 2023; Cui et al., 2020). In 2020, approximately 595 million people globally were living with OA, with knee OA accounting for more than 60% of cases and contributing to over 19 million years lived with disability (YLDs). The Global Burden of Disease Study projects a near-doubling of knee OA prevalence by 2050, driven by aging populations, rising obesity rates, and increasing joint injury incidence (Long et al., 2022). This escalating burden imposes enormous socioeconomic costs, exceeding $100 billion annually in high-income countries alone when direct medical expenses and lost productivity are combined (Hunter and Bierma-Zeinstra, 2019).

Current management of knee OA relies predominantly on symptomatic treatments. Oral analgesics and nonsteroidal anti-inflammatory drugs provide limited pain relief but are associated with significant gastrointestinal, cardiovascular, and renal adverse effects in elderly patients (Kolasinski et al., 2019). Intra-articular (IA) corticosteroids offer rapid but transient anti-inflammatory effects and may accelerate cartilage loss with repeated use (Jüni et al., 2015). Hyaluronic acid viscosupplementation yields modest and variable clinical benefits that typically dissipate within 6 months, while evidence for disease modification remains lacking (Bannuru et al., 2019). Emerging biologic approaches, including platelet-rich plasma and mesenchymal stem cell therapies, have shown inconsistent efficacy in rigorous trials and suffer from poor standardization (Nakagawa et al., 2025; Katz et al., 2021). Critically, no pharmacologic therapy is currently approved as a disease-modifying osteoarthritis drug (DMOAD), leaving total knee arthroplasty as the only definitive intervention for end-stage disease (Katz et al., 2021).

The failure of conventional intra-articular therapies to achieve sustained therapeutic concentrations within avascular cartilage, combined with rapid clearance from the synovial compartment (half-lives often <24 h), underscores the need for advanced drug delivery strategies (Maudens et al., 2018). Nanodelivery systems, sized 10–200 nm, exploit the unique physiology of the osteoarthritic knee to prolong joint residence, enhance penetration into dense cartilaginous matrices, and enable targeted delivery to specific cell populations or pathological microenvironments (DeJulius et al., 2021; Intini and O'Brien, 2026). Recent advances in surface functionalization, stimuli-responsive release, and cartilage-homing ligands have dramatically improved retention (from days to months) and achieved site-specific modulation of inflammation, catabolism, and matrix degradation in preclinical models (Wen et al., 2023; Rahimi et al., 2021).

This review critically examines the latest breakthroughs in precision-targeted intra-articular (IA) nanodelivery systems for knee OA, with particular emphasis on engineering strategies that overcome anatomical and pathophysiological barriers. We discuss stimuli-responsive and theranostic platforms, summarize progress from preclinical validation to early clinical translation, and identify key scientific, regulatory, and manufacturing challenges that must be addressed to realize clinically viable DMOADs.

## 2 Biological and mechanical barriers in the knee joint

OA is a complex, whole-joint disorder characterized by progressive degeneration of articular cartilage, subchondral bone remodeling, synovial inflammation, meniscal damage, ligamentous laxity, and periarticular muscle weakness (Hunter and Bierma-Zeinstra, 2019; Loeser et al., 2012). This multifactorial pathology is driven by bidirectional molecular crosstalk among joint tissues, resulting in a self-perpetuating cycle of mechanical overload, low-grade inflammation, and matrix degradation (Martel-Pelletier et al., 2016). Effective IA drug delivery must therefore contend not only with the avascular and densely packed nature of cartilage but also with dynamic clearance mechanisms and a hostile biochemical microenvironment that collectively limit therapeutic efficacy (Sanchez-Lopez et al., 2022; Hodgkinson et al., 2022).

### 2.1 Multi-tissue pathology of knee OA: a whole-joint disease

**FIGURE 1:**
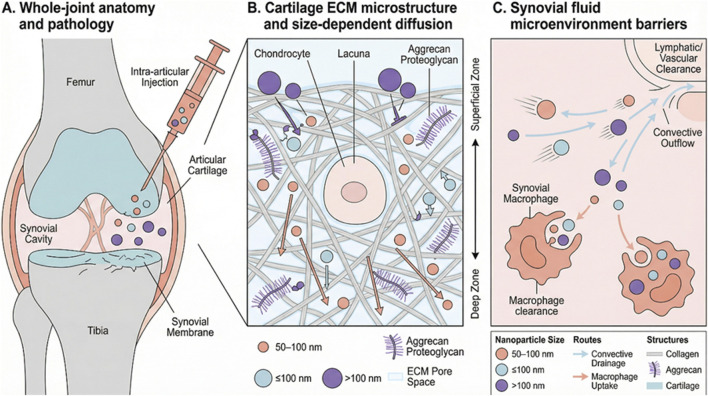
Whole-joint pathology and key anatomical, biomechanical and microenvironmental barriers to intra-articular nanodelivery in knee osteoarthritis. **(A)** Whole-joint anatomy and KOA-related pathology with IA injection into the synovial cavity. **(B)** Cartilage extracellular matrix (ECM) microstructure around a chondrocyte, showing collagen–aggrecan architecture, negatively charged glycosaminoglycan (GAG) chains, ECM pore space, and size-dependent diffusion (smaller particles penetrate deeper; larger particles are hindered). **(C)** Synovial fluid barriers, including convective outflow toward lymphatic/vascular clearance and synovial macrophage uptake, which reduce joint residence time and lesion exposure.

Contemporary understanding frames knee OA as a failure of the entire synovial joint rather than an isolated cartilage disease. Cartilage loss is accompanied by subchondral bone sclerosis, osteophyte formation, bone marrow lesions, and microarchitectural deterioration (Goldring and Goldring, 2010). Synovitis, detected histologically in >50% of early OA and nearly all advanced cases, correlates strongly with pain and structural progression (McHugh, 2017). Infrapatellar fat pad fibrosis and macrophage infiltration release proinflammatory mediators (IL-1β, TNF-α, IL-6) that amplify chondrocyte catabolism and inhibit anabolism (Cutolo et al., 2016). Meniscal extrusion and degeneration further exacerbate instability and accelerate cartilage erosion (Cañete et al., 2009). This interconnected pathology demands therapeutic platforms capable of simultaneous engagement with multiple tissue compartments. As schematically illustrated in Figure 1, knee osteoarthritis involves whole-joint pathology and multiple anatomical, biomechanical, and microenvironmental barriers that critically influence intra-articular nanodelivery.

### 2.2 Anatomical features of the knee joint relevant to drug delivery

The knee joint presents unique anatomical barriers. Articular cartilage is aneural, avascular, and alymphatic, with a highly organized extracellular matrix (ECM) comprising type II collagen fibrils (interfibrillar spacing ∼60–200 nm) enmeshed in a dense network of negatively charged aggrecan glycosaminoglycans (GAGs) (Sophia Fox et al., 2009). Fixed negative charges generate a Donnan partitioning effect that electrostatically repels anionic molecules while hindering diffusion of neutral or cationic species larger than ∼10 nm (Mlynárik and Trattnig, 2000). In OA, cartilage becomes hypocellular and fibrillated, yet the remaining ECM retains significant hindrance to macromolecular penetration (DiDomenico et al., 2018). The synovium, by contrast, is highly vascularized and permeable to particles up to several hundred nanometers, facilitating rapid systemic clearance via subsynovial lymphatics and blood capillaries (DiDomenico et al., 2018).

### 2.3 Synovial clearance and biomechanical factors affecting intra-articular retention

In the intra-articular (IA) compartment, residence time is governed not only by diffusion but also by synovial fluid turnover and clearance routes, including lymphatic drainage, vascular transport across the synovium, and sequestration by synovial macrophages. These pathways impose size- and surface-dependent elimination and interact with joint motion and shear to influence carrier integrity. Accordingly, IA nanocarriers should be designed around joint-specific clearance mechanisms rather than extrapolating from systemic pharmacokinetic assumptions.

Joint movement and load-bearing dramatically accelerate drug clearance. During gait, intra-articular pressure oscillates between subatmospheric and supraphysiologic values, driving convective fluid outflow through synovial capillaries and lymphatics (Georgiev, 2020; Martin et al., 2025). Small molecules (<500 Da) exhibit half-lives of minutes to hours, whereas macromolecules and microparticles (>1 µm) are cleared within days via phagocytosis or lymphatic drainage (Svirskis et al., 2018). Nanoparticles (NPs) in the 20–200 nm range achieve longer retention (weeks) owing to reduced phagocytosis and limited lymphatic uptake, but clearance remains highly sensitive to particle stiffness, shape, and surface properties (Brown et al., 2019). Elevated synovial fluid turnover in inflamed joints (up to 2–3 mL/h) further shortens residence time30.

### 2.4 Inflammatory microenvironment and biochemical triggers in KOA

The OA joint microenvironment is biochemically hostile. Synovial fluid pH drops to 6.6–7.2 in inflamed knees (versus ∼7.8 in healthy joints) due to lactic acid accumulation and impaired buffering (Hasnain et al., 2025; Loeser et al., 2016). Reactive oxygen species (ROS), including superoxide, hydrogen peroxide, and peroxynitrite, are markedly elevated and drive oxidative damage to cartilage matrix components (Henrotin et al., 2005). Catabolic enzymes—MMP-1, -3, -13, a disintegrin and metalloproteinase with thrombospondin motifs (ADAMTS) −4, and ADAMTS-5—are upregulated >10-fold, rapidly degrading collagen II and aggrecan (Mehana et al., 2019). Progressive GAG depletion reduces fixed negative charge density from ∼200 mEq/L in healthy cartilage to <100 mEq/L in advanced OA, altering electrostatic interactions with charged nanocarriers (Joshi et al., 2024). Hyaluronidase activity further fragments endogenous and exogenous hyaluronic acid, compromising lubrication and carrier stability (Duan et al., 2020).

### 2.5 Design implications for precision-targeted nanodelivery systems

These barriers dictate stringent design criteria for next-generation IA nanocarriers. Particle size should be optimized within 40–150 nm to maximize cartilage penetration while minimizing lymphatic clearance (Vedadghavami et al., 2019). Positively charged or cartilage-affinity ligands (e.g., WYRGRL peptide, collagen II-binding domains) exploit residual negative charge to achieve deep-zone penetration and chondrocyte internalization (Rothenfluh et al., 2008; Pi et al., 2011). Deformable or ultra-soft NPs resist mechanical expulsion under compressive and shear forces (Nia et al., 2013). Stimuli-responsive linkers—pH-sensitive hydrazones, ROS-cleavable thioketals, and matrix metalloproteinase (MMP) -degradable peptides—enable spatially and temporally controlled release within pathological niches while preserving payload in healthy tissue (Li et al., 2020; Ma et al., 2021). Integration of lubricating moieties and macrophage-repolarizing agents further addresses the whole-joint nature of OA, offering potential for synergistic disease modification (Chen et al., 2024; Yu et al., 2024).

## 3 Engineering principles of nanodelivery systems for intra-articular therapy

IA nanodelivery for OA hinges on rational design that simultaneously optimizes joint retention, cartilage penetration, payload release, and long-term biocompatibility. Recent advances have shifted the paradigm from passive sustained-release depots to actively targeted, stimuli-responsive, and multifunctional platforms capable of engaging specific cell populations and pathological microenvironments within the joint (Intini and O'Brien, 2026; Natesh et al., 2021).

### 3.1 Key physicochemical parameters

The physicochemical properties of NPs significantly influence their fate and performance in IA delivery, affecting retention, cartilage penetration, and clearance from the joint.

Particle size plays a crucial role in determining IA retention and tissue penetration. NPs smaller than 20 nm are prone to rapid lymphatic drainage and systemic leakage, whereas particles larger than 250 nm are quickly cleared by synovial macrophages within hours (Sheng et al., 2021). The optimal size range for sustained retention and effective cartilage penetration is between 40 and 150 nm. Ultrathin disk-shaped or filamentous carriers, particularly those with sizes around 10 nm, can achieve deeper zonal penetration due to reduced steric hindrance and enhanced diffusion through collagen II pores (Nia et al., 2013; Kim S-N. et al., 2021). In addition to size, particle stiffness has a profound impact on retention. Ultra-soft polyethylene glycol (PEG)-based nanogels with an elastic modulus of less than 1 kPa are less prone to compressive expulsion during joint loading, allowing for extended retention durations, ranging from several days to more than 4 weeks, compared to their stiffer counterparts (Kim et al., 2015).

Surface charge and hydrophilicity/hydrophobicity also play significant roles in IA delivery. Neutral or mildly cationic surfaces (zeta potential +5 to +25 mV) facilitate efficient cartilage uptake via electrostatic attraction to negatively charged proteoglycans while minimizing inflammation within the synovium (Boyer et al., 2025). However, excessive cationic charge (>+35 mV) can trigger macrophage activation and cytotoxicity, potentially leading to adverse effects (Lewinski et al., 2008). To mitigate these risks, PEGylation or zwitterionic coatings can be employed, which reduce protein corona formation and phagocytosis, extending joint residence times up to 28 days in rodent and equine models (Hu et al., 2018).

Biodegradability and the nature of degradation products are key factors influencing the safety and efficacy of nanocarriers. FDA-approved materials such as poly (lactic-co-glycolic acid) (PLGA), poly (ε-caprolactone), and hyaluronic acid (HA) are widely used due to their predictable hydrolysis rates and the non-toxic acidic byproducts they generate, which are buffered within the joint, making them suitable for clinical use (Makadia and Siegel, 2011). Additionally, polymers like poly (ortho esters) and poly (β-amino esters) enable surface-erosive degradation, providing sustained, near-zero-order release kinetics over extended periods, potentially up to several months (Palamoor et al., 2013). These properties make them ideal candidates for long-term therapeutic delivery in OA treatment.

### 3.2 Materials platforms for intra-articular nanodelivery

Intra-articular nanodelivery platforms play a crucial role in the targeted delivery of DMOADs, enabling efficient treatment of OA with enhanced therapeutic efficacy and reduced systemic side effects. Various material platforms have been explored to optimize the delivery of both small-molecule drugs and biologics to the joint tissue.

NPs, particularly poly (lactic-co-glycolic acid) (PLGA) and PEG-PLGA, remain the gold standard for encapsulating small-molecule DMOAD candidates, such as kartogenin and celecoxib, as well as nucleic acids (Ma et al., 2022). These NPs offer excellent drug-loading efficiency and stability. Amphiphilic block copolymer micelles, typically in the size range of 20–80 nm, self-assemble into nanocarriers with high drug-loading capacity (>15% w/w) and functionalizable surface coronas, making them suitable for targeted delivery and controlled release (Xu et al., 2021).

Lipid-based nanocarriers, including flexi-liposomes and elastic liposomes, incorporate edge activators like Tween 80, which enable the liposomes to deform under shear stress and improve cartilage penetration (Elsayed et al., 2006). Solid lipid nanoparticles (SLN) and nanostructured lipid carriers (NLC), such as those containing dexamethasone palmitate or triamcinolone acetonide hexacetonide (FX006, Zilretta®), have achieved clinical success, demonstrating 12-week pain relief in phase III trials (Conaghan et al., 2018).

Inorganic and hybrid nanomaterials also provide promising platforms for intra-articular delivery. Mesoporous silica nanoparticles (MSNs) functionalized with collagen II-targeting peptides enhance deep cartilage penetration and sustain the release of therapeutic agents, such as anti-IL-1β antibodies (Zong et al., 2023). Metal-organic frameworks (MOFs), loaded with compounds like baicalin, are used for macrophage repolarization through ROS scavenging, facilitating on-demand payload liberation (Li et al., 2024). Additionally, gold and cerium oxide nanozymes possess intrinsic superoxide dismutase (SOD) and catalase (CAT)-mimetic activity, helping to attenuate oxidative stress independently of their drug cargo (Cai et al., 2025).

**FIGURE 2:**
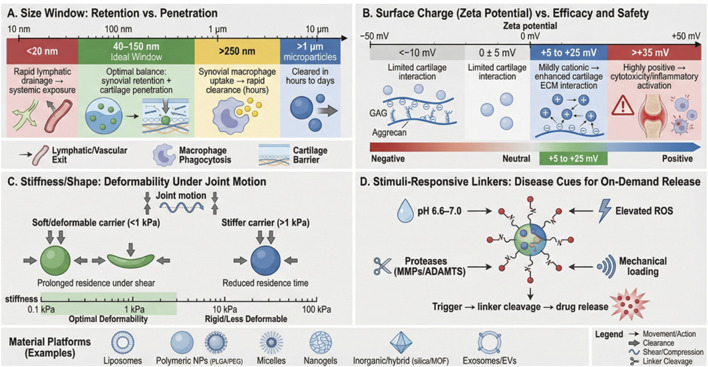
Design space of intra-articular nanocarriers for knee osteoarthritis: quantitative guidelines for key physicochemical parameters, material platforms, and stimuli-responsive linkers. **(A)** Size window trade-offs between retention and cartilage penetration: very small particles (<20 nm) are prone to lymphatic/vascular drainage and systemic exposure; an intermediate “ideal window” (∼40–150 nm) often balances synovial retention with cartilage access; larger particles (>250 nm) are more susceptible to synovial macrophage uptake and rapid clearance (hours); and microparticles (>1 μm) are typically cleared within hours to days. **(B)** Surface charge (zeta potential) versus efficacy and safety: mildly cationic carriers (∼+5 to +25 mV) can enhance electrostatic interaction with negatively charged cartilage ECM (GAG/aggrecan), whereas highly positive surfaces (>+35 mV) increase cytotoxicity and inflammatory activation; near-neutral or negative surfaces show limited cartilage interaction. **(C)** Stiffness/shape effects under joint motion: soft/deformable carriers (<1 kPa) better tolerate shear/compression and may prolong intra-articular residence, while stiffer carriers exhibit reduced deformability and shorter residence under mechanical stress. **(D)** Stimuli-responsive linkers enabling on-demand release using OA-relevant cues, including mild acidosis (pH ∼6.6–7.0), elevated reactive oxygen species (ROS), catabolic proteases (MMPs/ADAMTS), and mechanical loading, following a “trigger → linker cleavage → drug release” mechanism.

Bio-inspired nanocarriers, such as mesenchymal stem cell (MSC) -derived exosomes (50–150 nm), exhibit natural homing properties to inflamed synovium and cartilage, delivering therapeutic microRNAs (e.g., miR-100-5p, miR-140) that inhibit key matrix metalloproteinases (ADAMTS-5, MMP-13) involved in cartilage degradation (Qiu et al., 2020). Engineered exosomes displaying chondrocyte-affinity peptides further enhance targeting specificity (Wang et al., 2026). Additionally, self-assembling peptide amphiphiles form nanofibrous hydrogels *in situ*, providing both mechanical support and sustained release of biologics such as TGF-β3 or kartogenin (Liu et al., 2016). These diverse nanocarrier platforms represent the cutting edge of OA treatment, offering tailored strategies for effective intra-articular drug delivery. The overall design space of intra-articular nanocarriers, including size, stiffness, surface charge, stimuli responsiveness and representative material platforms, is summarized in Figure 2.

A comparative assessment of major IA nanocarrier classes across key performance attributes and translational considerations is provided in Table 1.

**TABLE 1:** Precision-targeted intra-articular nanodelivery strategies for knee osteoarthritis: design features, therapeutic mechanisms and translational considerations.

Strategy	Main target(s) in the knee	Representative nanoplatforms (examples)	Key features/Potential benefits in KOA
Cartilage ECM–targeted delivery	Negatively charged cartilage ECM (collagen II, aggrecan)	Cationic avidin nanoparticles; collagen II–binding peptide (e.g., WYRGRL)–modified PEG/PLGA NPs	Deep cartilage penetration and prolonged retention; improved local exposure of DMOADs with reduced systemic leakage
Chondrocyte-targeted delivery	OA chondrocytes (CD44, integrins, senescent cells)	HA-coated micelles/liposomes; ADAMTS-5 siRNA or CRISPR–Cas9 lipid NPs; senolytic-loaded NPs	Direct modulation of chondrocyte catabolism/anabolism and senescence; potential structural disease modification
Synovium/macrophage-targeted delivery	Inflamed synovium, M1-polarized macrophages, fibroblast-like synoviocytes	Tuftsin- or mannose-decorated NPs; folate-targeted dexamethasone liposomes; IL-4/IL-10–loaded PLGA NPs; ROS-scavenging nanozymes	Potent control of synovitis and cytokine release; M1→M2 macrophage repolarization; indirect protection of cartilage and subchondral bone
MSC-related and exosome-based platforms	Whole joint microenvironment (synovium, cartilage, subchondral bone)	Nanoengineered MSCs; MSC-derived exosomes carrying miR-140, miR-100-5p, miR-29 or lncRNA H19; vesicles embedded in thermosensitive hydrogels	Combined immunomodulatory and pro-regenerative effects; prolonged joint residence; possibility of phenotype-tailored nanotherapy
Subchondral bone and pain-targeted delivery	Subchondral osteoclasts; pain pathways (DRG neurons, TRPV1+)	Bisphosphonate-conjugated NPs delivering anti-resorptives; liposomal capsaicin; TRPV1-targeted siRNA NPs	Addresses abnormal bone remodeling and OA-related pain; complementary to cartilage-focused nanotherapies
Microenvironment-responsive (“smart”) systems	Acidic, ROS- and enzyme-rich, mechanically loaded OA niches	pH- and ROS-responsive dexamethasone or kartogenin NPs; MMP/ADAMTS-cleavable microgels; mechanosensitive carriers	On-demand, site-specific drug release in diseased regions; improved therapeutic index with reduced off-target toxicity
Lubricating and mechanically adaptive nanomaterials	Cartilage surface and joint lubrication layer	Bottle-brush polyelectrolyte NPs; phosphorylcholine-grafted NPs; dual-function lubricating/ROS-scavenging microgels	Rapid friction and wear reduction versus HA; simultaneous lubrication and attenuation of oxidative/inflammatory damage
Theranostic and clinical-stage nano (-like) depots	Diseased joint regions for imaging and sustained dosing	SPIO- or^19^F-labeled cartilage-targeting NPs (MRI/^19^F-MRI); NIR-II/photoacoustic theranostic NPs; triamcinolone acetonide extended-release microspheres (Zilretta®)	Image-guided tracking of joint retention and response; clinical proof-of-concept for sustained IA nano-/microformulations

Representative examples correspond to nanoplatforms and concepts discussed within this review, rather than an exhaustive list of all published systems. ADAMTS, a disintegrin and metalloproteinase with thrombospondin motifs; DMOAD, disease-modifying osteoarthritis drug; DRG, dorsal root ganglion; ECM, extracellular matrix; GAG, glycosaminoglycan; HA, hyaluronic acid; IA, intra-articular; IL-1Ra, interleukin-1, receptor antagonist; KOA, knee osteoarthritis; MMP, matrix metalloproteinase; MSC, mesenchymal stem cell; NIR, near infrared; NLRP3, NOD-, LRR- and, pyrin domain–containing protein 3; NP, nanoparticle; OA, osteoarthritis; ROS, reactive oxygen species; SASP, senescence-associated secretory phenotype; SPIO, superparamagnetic iron oxide.

### 3.3 Stimuli-responsive release strategies

In recent years, stimuli-responsive release strategies have garnered significant attention for targeted drug delivery in the treatment of OA. These strategies rely on the exploitation of specific triggers, such as pH, ROS, redox environments, enzymes, and mechanical forces, to achieve selective and controlled therapeutic release.

pH-responsive nanoplatforms utilize linkers such as hydrazone, acetal, and cis-aconityl, which undergo cleavage at the synovial pH range of 6.6–7.0, selectively releasing therapeutic agents in inflamed joints while remaining stable under normal physiological conditions (pH 7.4) (Zhang J. et al., 2021). ROS- and redox-responsive systems, on the other hand, exploit thioketal, arylboronic ester, and disulfide bonds, which are cleaved by elevated levels of hydrogen peroxide (H_2_O_2_) and glutathione in OA cartilage, resulting in a significantly higher release rate in diseased tissue compared to healthy tissue, often exceeding a 10-fold increase (Ma et al., 2021; Zhang H. et al., 2021). Enzyme-responsive and matrix-degradable carriers utilize peptide sequences, such as GPLGLAGQ, which are cleaved by matrix metalloproteinases (MMP-2/9) or ADAMTS, present during active matrix degradation, ensuring that drug release is synchronized with peak catabolic activity (Wu et al., 2025). Additionally, mechanically responsive systems leverage shear- and compression-sensitive linkers, such as mechanolabile azo-bonds, or shape-memory polymers to trigger drug release during weight-bearing activities, aligning therapeutic delivery with symptomatic episodes (Wu et al., 2025). These approaches offer promising solutions for the targeted treatment of OA, addressing the need for precise and controlled release mechanisms in response to disease-specific conditions.

### 3.4 Safety and biocompatibility considerations in intra-articular nanodelivery

When developing nanomaterials for IA drug delivery, it is crucial to carefully consider the potential for chondrotoxicity, immune responses, and systemic exposure, as these factors directly impact both the safety and therapeutic efficacy of the treatment.

Chondrotoxicity is a primary concern, particularly with high-dose cationic NPs exceeding 100 μg/mL. At these concentrations, NPs can disrupt chondrocyte membrane integrity and induce apoptosis through mitochondrial ROS generation (Yan et al., 2025). However, PEGylated neutral or mildly cationic NPs, when administered at therapeutic doses below 50 μg/mL, have been shown to preserve GAG synthesis and maintain chondrocyte viability in human cartilage explants for up to 28 days, indicating their potential for safe, long-term use in clinical applications (Huang K. et al., 2025).

In terms of immune responses, repeated IA injections of non-degradable particles can provoke foreign-body reactions and cause persistent synovitis, which may lead to long-term complications (Dong et al., 2020). Biodegradable lipid and polymeric nanocarriers, on the other hand, generally elicit minimal innate immune activation. Notably, when functionalized with anti-inflammatory agents like IL-4 or dexamethasone, these systems can promote macrophage polarization toward M2 phenotypes, enhancing anti-inflammatory responses and minimizing tissue damage (Yuan et al., 2025).

Systemic exposure and off-target organ accumulation are also critical safety concerns. For optimized 80–150 nm PEGylated NPs, systemic exposure is consistently below 5% of the injected dose in large-animal models, suggesting that these particles can be effectively confined to the joint with minimal off-target effects. Furthermore, NPs smaller than 10 nm are primarily cleared via renal pathways, while hepatic and splenic uptake is negligible for biodegradable platforms, ensuring that these carriers do not accumulate in non-target organs (Li et al., 2021). These findings highlight the importance of selecting the right particle size and biodegradability to minimize adverse systemic effects while maximizing local therapeutic benefits in OA treatment.

## 4 Precision-targeted nanodelivery strategies in knee osteoarthritis

To improve clarity, we distinguish three complementary layers of precision targeting in IA nanodelivery for KOA: (i) passive retention strategies (e.g., size/shape/stiffness tuning and depot-forming carriers): that prolong synovial residence; (ii) active ligand-/receptor-mediated targeting to specific joint tissues or cell populations (e.g., cartilage ECM, chondrocytes, synovium, and inflammatory macrophages); and (iii) stimuli-responsive systems that leverage disease-associated cues (pH, ROS, proteases, or mechanical loading) to trigger on-demand release.

**FIGURE 3:**
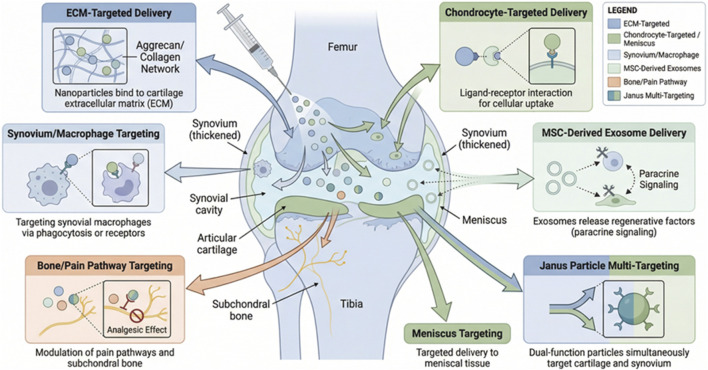
Spatial map of precision-targeted intra-articular nanodelivery strategies within the osteoarthritic knee joint. Figure 3 depicts the anatomical structure of the osteoarthritic knee joint (femur, synovium, articular cartilage, meniscus, etc.) and demonstrates nanodelivery strategies targeting different sites: including ECM-targeted delivery, chondrocyte-targeted delivery, synovium/macrophage-targeted delivery, MSC-derived exosome delivery, bone/pain pathway targeting, and Janus particle-mediated multi-target delivery systems, reflecting the precision and multi-dimensional coverage of local intra-articular drug delivery.

The heterogeneous pathology of knee OA necessitates precision targeting beyond simple prolonged retention. Recent efforts have focused on ligand-guided, charge-based, and cell-specific nanocarriers that selectively accumulate in diseased cartilage, chondrocytes, inflamed synovium, or subchondral bone, thereby maximizing therapeutic index while minimizing off-target effects (Brown et al., 2019; Bajpayee and Grodzinsky, 2017). Figure 2 focuses on the physicochemical prerequisites for optimized delivery, while Figure 3 provides the spatial mapping of these strategies within the OA joint. Figure 3 provides a spatial map of these precision-targeted intra-articular nanodelivery strategies within the osteoarthritic knee joint.

### 4.1 ECM-targeted nanodelivery

Effective targeting and distribution of nanomaterials within the cartilage ECM are essential for optimizing therapeutic outcomes in OA treatment. Several strategies have been developed to enhance the selective delivery and penetration of nanocarriers into the cartilage tissue.

Charge-mediated targeting exploits electrostatic interactions between positively charged nanocarriers and the negatively charged proteoglycans present in the cartilage ECM. For example, avidin, with a zeta potential of approximately +28 mV, and guanidinium-rich cationic peptides, exhibit rapid penetration of full-thickness cartilage within 24 h, achieving more than 100-fold higher uptake compared to neutral or anionic nanoparticles (He et al., 2020). Cationic dendrimers conjugated with dexamethasone have also shown prolonged drug retention in cartilage for over 30 days in rat and rabbit post-traumatic OA (PTOA) models, leading to a 45%–60% reduction in OARSI scores (Geiger et al., 2018).

Ligand-based targeting further enhances nanocarrier specificity to the cartilage ECM components. Peptides such as WYRGRL, which binds to collagen IIα1, and DWRVIIPPRPSA, which binds to aggrecan, when conjugated to PEG or PLGA NPs, significantly increase the accumulation of NPs in deep cartilage zones (Geiger et al., 2018). Additionally, a 14-mer collagen II-homing peptide (RRTRR) linked to gold NPs demonstrates six times higher retention in damaged versus intact cartilage in equine explants (Rothenfluh et al., 2008; Pi et al., 2011). Chondroitin sulfate-binding peptides can further enhance binding to regions of the ECM enriched in exposed epitopes, particularly in degraded cartilage areas (Zhu et al., 2022).

To improve deep-zone penetration and spatial distribution, size and mechanical properties of NPs are critical. NPs smaller than 40 nm and with an ultra-soft Young’s modulus of less than 1 kPa can overcome steric and electrostatic barriers, achieving uniform distribution throughout the superficial to deep zones of cartilage (Vedadghavami et al., 2019). Additionally, “Donnan partitioning,” which restricts the penetration of larger particles, can be temporarily neutralized by co-injecting high-osmolarity saline, enabling even larger (greater than 100 nm) PEGylated NPs to effectively penetrate cartilage tissue *in vivo* (Bajpayee et al., 2017). These strategies are instrumental in enhancing the localization and therapeutic effectiveness of nanocarriers in OA treatment.

### 4.2 Chondrocyte-targeted nanodelivery

Receptor-mediated targeting strategies have proven to be effective in enhancing the specificity and efficacy of IA drug delivery for OA, particularly in addressing the unique cellular and molecular characteristics of the joint.

In OA, chondrocytes overexpress CD44, a receptor that plays a key role in cell signaling and ECM interactions. Hyaluronic acid (HA)-coated liposomes and micelles, which bind to CD44 with nanomolar affinity, facilitate internalization via receptor-mediated endocytosis, making them effective delivery vehicles for targeted therapeutics (Cai et al., 2017). Additionally, peptides that bind α10β1 integrins, such as AEDG, conjugated to lipid NPs, enhance chondrocyte uptake by 12-fold, while also preserving GAG content in IL-1β-stimulated cartilage explants, demonstrating the potential for preserving cartilage integrity in inflammatory environments (Miao et al., 2024).

Nanocarriers are also being explored for the delivery of DMOADs, nucleic acid therapeutics, and gene-editing cargos. Kartogenin-loaded HA-micelles have been shown to promote chondrogenesis and significantly reduce Mankin scores by 50% in anterior cruciate ligament transection (ACLT) rat models of OA (He et al., 2025). Moreover, PEG-avidin/siRNA complexes targeting ADAMTS-5, a key enzyme involved in cartilage degradation, can silence gene expression for up to 28 days in murine cartilage, effectively preventing GAG loss and supporting cartilage preservation (Jiang et al., 2021). CRISPR-Cas9 ribonucleoproteins delivered via collagen II-targeted lipid NPs have demonstrated a 35% editing efficiency in human OA chondrocytes *ex vivo*, suggesting a promising approach for genetic interventions in OA treatment (Liang et al., 2022).

Beyond drug delivery, nanocarriers also offer the potential to modulate chondrocyte catabolism, anabolism, senescence, and apoptosis, key processes involved in OA pathogenesis. Nanocarriers co-delivering NF-κB inhibitors and SOX9 plasmids have been shown to restore the anabolic/catabolic balance in cartilage, promoting tissue repair and reducing inflammation (Huang H. et al., 2025). Additionally, senolytic-loaded NPs selectively target and clear p16INK4a-positive senescent chondrocytes in post-traumatic OA models, effectively reducing the senescence-associated secretory phenotype (SASP) and delaying OA progression (Gil et al., 2022). These advances in receptor-mediated targeting and nanocarrier-based delivery hold great promise for developing more effective and personalized treatments for OA, addressing both the symptoms and underlying mechanisms of the disease.

### 4.3 Synovium- and macrophage-targeted nanodelivery

Nanocarriers targeting synoviocytes, macrophages, and other immune cells have emerged as promising strategies for modulating the inflammatory microenvironment of OA, providing targeted therapeutic interventions to address joint inflammation and cartilage degradation.

Tuftsin-modified liposomes and mannose-conjugated polymeric micelles have been designed to selectively bind activated synoviocytes, including macrophage-like synoviocytes and fibroblast-like synoviocytes. These nanocarriers efficiently deliver therapeutic agents such as methotrexate or p65 siRNA, suppressing synovial hyperplasia and inflammation, which is a hallmark of OA progression (You et al., 2018). This targeted approach helps reduce the systemic side effects often associated with traditional therapies, focusing the treatment on the inflamed joint tissue.

In addition to targeting synoviocytes, nanoplatforms have been developed to modulate macrophage polarization, which plays a pivotal role in OA pathogenesis. M1 macrophages, typically pro-inflammatory, are characterized by the overexpression of folate receptor-β, while M2 macrophages, associated with tissue repair and anti-inflammatory functions, express mannose receptors. Nanocarriers targeting these receptors can selectively repolarize macrophages. Folate-targeted dexamethasone liposomes have been shown to shift the M1→M2 phenotype, significantly reducing levels of pro-inflammatory cytokines such as IL-1β and TNF-α in the synovial fluid by more than 70% in collagen-induced arthritis models (Song et al., 2021). Similarly, PLGA NPs dual-loaded with IL-4 and IL-10, key cytokines that promote M2 polarization, have been shown to attenuate synovitis and cartilage erosion in the K/BxN serum-transfer arthritis model, demonstrating their potential in modulating the immune response to prevent OA progression (Li et al., 2022).

To further enhance immune regulation in OA, immunomodulatory nanotherapies, such as ROS-scavenging cerium oxide nanozymes and bilirubin-conjugated NPs, have been developed to suppress (NOD-, LRR- and pyrin-domain–containing protein 3) NLRP3 inflammasome activation. This pathway plays a critical role in IL-1β maturation and pyroptosis, which contribute to chronic inflammation in OA. These NPs help mitigate the inflammatory response by reducing the activation of NLRP3, leading to decreased IL-1β secretion and alleviating synovial macrophage-induced inflammation59. These approaches highlight the potential of nanomedicine to not only deliver therapeutics but also to directly modulate the immune environment of the joint, offering a multifaceted strategy for treating OA.

### 4.4 MSC-related and exosome-based nanoplatforms

Nanotechnology offers significant advancements in enhancing the survival, retention, and therapeutic potential of MSCs and their derivatives, providing promising strategies for OA treatment.

To improve MSC survival and retention in the joint, various nanotechnological approaches have been explored. For instance, iron oxide-labeled MSCs coated with an anti-inflammatory peptide, KAFAK, exhibit a five-fold increase in IA retention and enhanced secretion of anti-inflammatory cytokines such as TSG-6 and prostaglandin E2 (PGE2), both of which play crucial roles in modulating inflammation and promoting tissue repair (Le et al., 2020). Additionally, MSC-derived nanovesicles, when loaded into temperature-sensitive hydrogels, demonstrate greater than 90% retention in rabbit models for up to 28 days post-injection, providing sustained therapeutic effects (Zhang J. et al., 2025). These strategies enhance the longevity and efficacy of MSC-based therapies in OA, ensuring that therapeutic agents are retained in the joint for an extended period.

MSC-derived exosomes, which are nanosized extracellular vesicles, have also emerged as potent nanocarriers for delivering therapeutic cargos. Exosomes engineered with chondrocyte-affinity peptides, such as lamp2b-CART, show promising cartilage-homing capabilities. These exosomes effectively deliver miR-140, restoring the expression of collagen II (COL2A1) and inhibiting ADAMTS-5, a key enzyme involved in cartilage degradation (Liu et al., 2024). Moreover, exosomal delivery of long non-coding RNA (lncRNA) H19 has been shown to inhibit Wnt/β-catenin signaling, a pathway associated with OA progression, thus delaying disease progression in rat models (Behera et al., 2021). These findings highlight the potential of exosome-based therapies for targeted, gene-regulatory treatments in OA.

Further advances in MSC-related nanotherapeutics focus on targeting specific OA phenotypes. Exosomes derived from MSCs overexpressing miR-100-5p selectively attenuate the inflammatory phenotypes of OA, reducing synovial inflammation and cartilage degradation. On the other hand, miR-29-transfected exosomes are more effective in preserving cartilage in metabolic OA models, demonstrating the ability to tailor exosome-based therapies to different OA phenotypes (Kim GB. et al., 2021). These targeted approaches offer personalized treatment strategies, enhancing the therapeutic outcomes by addressing the specific molecular drivers of OA in individual patients.

Together, these innovations in MSC-related nanotherapeutics hold great promise for advancing OA treatment by improving cell retention, enhancing cartilage repair, and providing targeted, phenotype-specific therapeutic interventions.

### 4.5 Emerging targets and multi-target approaches

Nanotechnology is advancing the development of multi-faceted therapeutic strategies for OA by enabling targeted drug delivery to specific tissues, modulating pain pathways, and addressing multiple disease components simultaneously.

Nanodelivery to the subchondral bone and osteochondral units has been explored as a means to address the altered bone remodeling that characterizes OA. Bisphosphonate-conjugated NPs, which accumulate in osteoclast-rich subchondral regions, are designed to deliver drugs such as alendronate or cathepsin K inhibitors. These treatments help suppress aberrant bone remodeling, a key feature of OA, thereby slowing disease progression and improving joint function (Farrell et al., 2018).

Pain management in OA is another critical area of focus, particularly targeting neurosensory components involved in pain signaling. Liposomal formulations of capsaicin and TRPV1-targeted siRNA NPs have shown promise in reducing dorsal root ganglion sensitization, a key mechanism in OA-associated pain. These therapies reduce pain behaviors in monosodium iodoacetate (MIA)-induced OA rat models, providing an effective means to alleviate pain in OA patients while potentially minimizing the side effects of traditional pain management therapies (Lv et al., 2021).

Finally, the development of multi-target and whole-joint nanotherapeutic approaches is gaining traction for addressing the complex, multifactorial nature of OA. “Janus” NPs, which present both cationic faces for cartilage penetration and anionic faces for macrophage-repolarization, offer a dual mechanism to both protect cartilage and resolve synovitis by repolarizing pro-inflammatory macrophages to anti-inflammatory phenotypes (Liu Y. et al., 2023). Similarly, thermoresponsive hydrogel-embedded multifunctional NPs have been developed to achieve sequential drug release: an initial rapid burst of anti-inflammatory agents followed by the sustained delivery of DMOADs. This approach allows for the timely and controlled release of therapeutic agents, offering a tailored treatment strategy that addresses both inflammation and cartilage degeneration in OA (Deloney et al., 2020).

These innovative nanotherapeutic concepts highlight the potential of nanotechnology to provide more effective, targeted, and comprehensive treatments for OA, addressing the diverse aspects of the disease, from subchondral bone remodeling to pain management and immune modulation.

## 5 Stimuli-responsive and theranostic nanoplatforms for knee OA

**FIGURE 4:**
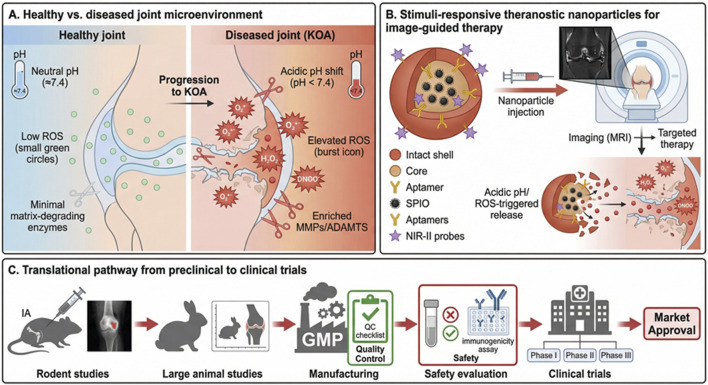
Stimuli-responsive and theranostic intra-articular nanoplatforms for knee osteoarthritis and their translational pathway. **(A)** Comparison of healthy versus KOA joint microenvironments, highlighting a shift from near-neutral pH (∼7.4), low reactive oxygen species (ROS), and minimal matrix-degrading enzymes in healthy joints to mild acidosis, elevated ROS, and enriched catabolic proteases (e.g., MMPs/ADAMTS) in diseased joints. **(B)** Concept of stimuli-responsive theranostic nanoparticles for image-guided therapy after intra-articular (IA) injection, illustrating multifunctional assemblies incorporating imaging components (e.g., SPIO and NIR-II probes) and targeting ligands (e.g., aptamers), enabling MRI-based monitoring and disease-cue-triggered activation/release (acidic pH/ROS) to enhance lesion-localized treatment. **(C)** Translational pathway from preclinical studies to clinical implementation, progressing from rodent proof-of-concept to large-animal validation, followed by GMP manufacturing and quality control, safety evaluation (including immunogenicity testing), and phased clinical trials toward market approval.

The hostile and dynamic intra-articular microenvironment of the osteoarthritic knee provides unique biochemical and biomechanical cues that can be harnessed for “smart” nanoplatforms. These systems remain inert in healthy joints but undergo programmed activation in response to OA-specific triggers, thereby achieving spatially and temporally controlled drug release, lubrication restoration, or image-guided therapy with unprecedented precision (Jiang and Zhang, 2023; Ye et al., 2024). Key precision-targeted intra-articular nanodelivery strategies, representative platforms and translational considerations are summarized in Table 2. Figure 4 further illustrates the OA versus healthy joint microenvironment, the functions of theranostic intra-articular nanoplatforms, and the stepwise translational pathway from rodent and large-animal models to human clinical trials.

**TABLE 2:** Comparative assessment of major intra-articular nanocarrier classes for knee osteoarthritis.

Nanoplatform class	Synovial retention	Cartilage penetration	Targeting efficiency options	Drug loading/Co-delivery	Biodegradability and safety	Clinical feasibility and scalability
Liposomes (incl. Elastic/stealth)	Medium	Medium	Medium (ligand/charge)	Medium	Medium	High
Polymeric NPs (e.g., PLGA/PEG-PLGA)	Medium-high	Medium	Medium-high (ligand/charge)	High	High (biodegradable)	Medium-high
Polymeric micelles	Low-medium	High	Medium	Medium	Medium-high	High
Nanogels/injectable hydrogels (depots)	High	Low-medium	Low-medium	High	Medium-high	Medium
Inorganic/hybrid (MSN, MOF, nanozymes)	Medium	Medium	Medium	High	Variable (depends on core)	Medium
Exosomes/engineered EVs	Medium	Medium-high	High (intrinsic tropism)	Medium	Medium-high	Low-medium

Ratings are qualitative and context-dependent; actual performance depends on formulation (size/charge/stiffness), disease stage, and evaluation methods. Clinical feasibility reflects CMC/GMP, complexity, sterilization constraints, batch reproducibility, and cost.

### 5.1 Microenvironment-responsive nanoplatforms in inflamed OA joints

Synovial pH in active OA drops to 6.4–6.8, while ROS levels rise >10-fold. pH-sensitive hydrazone or acetal linkages conjugated to dexamethasone prodrugs cleave selectively in inflamed joints, yielding 15-fold higher local drug concentrations than non-responsive controls in rat ACLT models (Su et al., 2021). ROS-responsive thioketal-linked dexamethasone dimeric prodrugs self-assemble into 90 nm NPs that release active drug only in the presence of H_2_O_2_, reducing cartilage degradation by 68% and synovitis by 75% in PTOA mice (Zhou et al., 2023). Dual pH/ROS-responsive PEG-b-poly (carbonate) NPs co-delivering kartogenin and rapamycin achieve synergistic chondroprotection and immunomodulation for over 8 weeks (Fan et al., 2024).

MMP-2/9- and ADAMTS-5-cleavable peptide sequences (e.g., GPVGLIGK or GPLGLAGQ) are widely incorporated as linkers between carrier and payload. MMP-responsive PEG microgels encapsulating TGF-β3 release growth factor proportionally to local enzyme activity, preserving 42% more GAG in IL-1β-stimulated human cartilage explants than constitutive-release controls (Yao et al., 2018). Enzyme-triggered charge-reversal NPs switch from anionic (stealth) to cationic (cartilage-penetrating) only in degraded ECM regions, achieving deep-zone accumulation in advanced OA (He et al., 2023).

### 5.2 Mechanically adaptive and lubricating nanomaterials

Bottle-brush polyacrylamide-sulfobetaine methacrylate NPs reduce the coefficient of friction of cartilage surfaces from 0.25 to <0.05 while simultaneously releasing diclofenac for 21 days (Lakin et al., 2019). Phosphorylcholine-grafted gold NPs form boundary lubricant films that withstand 10^6^ cycles of physiological loading without wear, outperforming HA viscosupplements in bovine explants (Pu et al., 2014).

Dual-function microgels combining glycerol monooleate liquid crystals (lubrication) and ROS-scavenging cerium oxide cores reduce friction by 80% and oxidative stress by 70% in rat MIA models, resulting in OARSI scores comparable to healthy controls at 12 weeks (Deng et al., 2025). Bioinspired “mucus-like” glycoprotein-mimetic NPs restore boundary lubrication while delivering IL-1Ra, synergistically attenuating pain and structural damage (Xie et al., 2021).

### 5.3 Theranostic nanoplatforms: integrating imaging and therapy

Superparamagnetic iron oxide (SPIO)-labeled, collagen II-targeted NPs enable T2-weighted MRI visualization of cartilage retention up to 28 days post-injection in rabbit ACLT models (Antonelli and Magnani, 2022). ^19^F-Perfluorocarbon nanoemulsions conjugated to WYRGRL peptide provide background-free ^19^F-MRI hotspot tracking of inflammation and simultaneous delivery of triamcinolone (Wang et al., 2021). Near-infrared-II fluorescent aggrecanase-activatable probes embedded in HA NPs allow real-time optical monitoring of disease activity and on-demand release of anti-ADAMTS-5 siRNA (Zhang et al., 2022).

Photoacoustic-responsive gold nanorods coated with black phosphorus and kartogenin trigger drug release upon near-infrared laser irradiation, achieving spatiotemporal control and 55% reduction in Mankin scores in rat PTOA (Chen and Liao, 2023). Ultrasound-triggered perfluorocarbon nanodroplets undergo acoustic droplet vaporization to enhance cartilage penetration of co-delivered IGF-1, doubling anabolic response in equine explants (Pu et al., 2024).

Multimodal (MRI/optical) NPs discriminating inflammatory versus mechanical OA phenotypes via differential synovial versus cartilage uptake enable patient stratification in preclinical large-animal studies (Zhan et al., 2024). Longitudinal theranostic monitoring correlates imaging signal decay with histological improvement, providing early surrogate endpoints for future clinical trials (Walther et al., 2023).

## 6 From preclinical models to clinical translation

The journey from preclinical studies to clinical translation of IA nanodelivery for knee OA is complex and involves multiple stages of evaluation, ranging from proof of concept to safety and efficacy trials. Despite promising outcomes in animal models, significant challenges remain in bridging the gap to human applications. Preclinical models are essential for evaluating pharmacokinetics, biodistribution, and therapeutic outcomes, yet translating these findings into human trials requires careful consideration of anatomical, biomechanical, and immune-system differences between species.

### 6.1 Animal models used for evaluating intra-articular nanotherapies

Rodent models, including the surgically induced destabilization of the medial meniscus (DMM) and monosodium iodoacetate (MIA) injection models, are widely used for studying the efficacy of IA nanodelivery systems. The DMM model induces progressive OA that closely mimics human knee joint degeneration, with alterations in cartilage, synovium, and subchondral bone (Glasson et al., 2007). The MIA model, which chemically induces cartilage degeneration by inhibiting glycolysis, is commonly used for pain-related studies due to the development of both mechanical and thermal hyperalgesia (Chung et al., 2010). Nanocarriers administered in these models have shown significant improvements in joint retention, drug release profiles, and therapeutic outcomes. For example, PLGA-based NPs loaded with anti-inflammatory agents significantly reduce synovial inflammation and cartilage degradation in MIA-injected rats (Pontes et al., 2022). Moreover, the long-term safety and immune response to these NPs are also typically assessed in these models, helping refine formulations before advancing to larger animal models.

Larger animal models, such as rabbits, dogs, and non-human primates, are increasingly being used to assess the clinical relevance of IA nanotherapy due to their closer anatomical and physiological similarities to humans. The rabbit anterior cruciate ligament transection (ACLT) model and the dog meniscectomy model are frequently employed to evaluate the therapeutic effects of IA nanocarriers on cartilage repair and functional recovery (Naba, 2020). In large animal studies, IA injection of MSC-derived exosome-based NPs and therapeutic siRNA-loaded particles has shown improved cartilage regeneration, reduced inflammation, and better joint function recovery compared to controls (Kang et al., 2021; Lu et al., 2025). Importantly, these studies are also pivotal for assessing the distribution of NPs in synovial joints, their long-term retention, and any potential adverse effects, such as chondrotoxicity or immune reactions.

### 6.2 Pharmacokinetics and biodistribution of nanocarriers in the knee compartment

The pharmacokinetics of IA nanocarriers depend on multiple factors, including particle size, surface charge, and the biochemical environment of the joint. Following IA injection, nanocarriers are subject to rapid clearance through synovial fluid turnover, lymphatic drainage, and macrophage uptake. Studies have shown that particles in the size range of 40–150 nm exhibit prolonged retention within the joint and greater tissue penetration, compared to smaller (under 20 nm) or larger particles (>250 nm), which are cleared more rapidly (Wen et al., 2023; Zhang T. et al., 2025). The retention time of these NPs is also influenced by the inflammatory state of the joint, with OA-induced alterations in synovial fluid dynamics and ECM composition promoting the retention of certain nanocarriers (Lawson et al., 2021). Additionally, imaging studies, such as MRI or fluorescence tracking, have been used to track the distribution and clearance of NPs in preclinical models. For instance, iron oxide NPs conjugated with cartilage-targeting peptides allow MRI-based monitoring of particle retention for up to 6 weeks, offering insights into the kinetics of drug delivery and clearance from the knee compartment (Kronthaler et al., 2022).

Although IA administration targets the knee joint, systemic leakage of NPs can occur, particularly for those that are not sufficiently “stealth” or biodegradable. This leakage is typically quantified by measuring the concentration of NPs in systemic circulation, tissues, and organs (e.g., liver, spleen, kidneys). Systemic exposure is often minimized by surface modification, such as PEGylation, which reduces macrophage uptake and prolongs circulation time (Delbaldo et al., 2022). However, even with such modifications, the potential for nanoparticle accumulation in off-target organs remains a concern. Recent studies on PLGA and lipid-based NPs have demonstrated systemic exposure is consistently below 5% of the injected dose in large-animal models when using appropriately sized particles (50–150 nm) and biodegradable materials (Gambaro et al., 2021). The systemic toxicity of NPs remains a critical consideration, and comprehensive evaluations of organ toxicity, particularly liver and kidney function, are crucial for determining the safety profiles of nanotherapies in clinical settings.

### 6.3 Representative preclinical studies of precision-targeted nanodelivery

Recent advances in preclinical studies have highlighted the potential of precision-targeted nanodelivery systems to not only reduce pain but also modify the disease process in knee OA. For example, collagen II-targeted NPs loaded with celecoxib have shown improved cartilage penetration and long-lasting anti-inflammatory effects, significantly reducing histological damage in DMM and MIA models (Shin et al., 2020). Similarly, gene-editing NPs carrying siRNA targeting ADAMTS-5 or MMP-13 have demonstrated up to 40% preservation of GAG content in cartilage, which could potentially slow the progression of OA (Duan et al., 2021). These studies provide robust evidence of the efficacy of IA nanocarriers in preclinical models and suggest that such treatments could lead to sustained therapeutic benefits, even in advanced stages of OA.

### 6.4 Early clinical experience and ongoing trials of nano- or nano-like intra-articular products

While preclinical evidence for IA nanodelivery systems is promising, clinical translation remains in its early stages. Several phase I and II clinical trials have been initiated to evaluate the safety and efficacy of nanoparticle-based therapies for OA. For instance, the intra-articular injection of triamcinolone acetonide-loaded NPs (Zilretta®) has shown efficacy in reducing knee pain and inflammation for up to 12 weeks, with no significant adverse effects in clinical trials (Liao et al., 2024). Other nanoparticle formulations under investigation include those delivering gene therapy or anti-inflammatory drugs. Early results indicate that these therapies may offer superior duration of action and fewer side effects compared to traditional injectable treatments like corticosteroids and hyaluronic acid (Mohammadinejad et al., 2020). However, challenges remain in standardizing formulations, ensuring reproducible manufacturing, and addressing patient variability in terms of OA phenotype and stage.

### 6.5 Lessons learned: discrepancies between animal models and human knee OA

Despite significant advances in preclinical models, there are notable discrepancies when translating these findings to human knee OA. While rodent models effectively replicate the cellular and molecular changes in OA, they do not fully mimic the mechanical and physiological aspects of human joints, such as load-bearing and long-term degradation. Furthermore, large animal models, despite their closer resemblance to human knee anatomy, still exhibit differences in joint biomechanics and inflammatory response that can influence the outcomes of nanomedicine treatment. These discrepancies highlight the need for improved human-relevant models and better patient stratification in clinical trials. In particular, the role of comorbidities, joint alignment, and muscle strength, which are often underrepresented in animal models, must be carefully considered in clinical studies to ensure the therapeutic efficacy of nanomedicines across different OA phenotypes.

## 7 Translational challenges and future directions

The translation of precision targeted IA nanodelivery systems for KOA into clinical reality remains encumbered by scientific, technical, regulatory, and clinical trial design hurdles. Acknowledging these barriers is essential to bridge the gap between promising preclinical data and approved therapies.

### 7.1 Scientific and technical challenges

Aligning nanocarrier design with the complex microenvironment of the osteoarthritic knee joint is deceptively difficult. As preclinical work has revealed, the cartilage ECM, synovium, subchondral bone, and joint fluid all impose distinct physicochemical and biological constraints on nanoparticle disposition and efficacy (Liu L. et al., 2023). For example, NPs optimized for cartilage penetration may perform suboptimally in targeting synovial fibroblasts or subchondral bone. The trade off between prolonged IA retention and efficient tissue penetration remains unresolved: carriers achieving months long retention often reside predominantly in synovial fluid or superficial cartilage rather than deep zone target sites (Liao et al., 2024). Moreover, the balance between sufficient payload release and acceptable carrier persistence is delicate; long lived materials raise concerns about chronic joint exposure and potential toxicity (Pontes et al., 2022).

From a manufacturing perspective, scale up of complex multifunctional nanocarriers (ligand‐modified, stimuli responsive, hybrid inorganic/organic) poses reproducibility and batch to batch consistency issues. Characterisation methods for size, shape, deformability, surface chemistry, and in joint behaviour are still evolving and are yet to be standardised for IA applications. While many studies demonstrate efficacy in rodent models, translating to large animal joints and ultimately human knees introduces variables in size, loading, motion, joint fluid volume, and clearance pathways that are seldom captured in early work (Lawson et al., 2021).

### 7.2 Regulatory and manufacturing barriers

The regulatory classification of IA nanodelivery systems often falls into a grey zone: drug, device, biologic, or combination product, depending on carrier composition, payload type, and delivery mechanism. Regulators require detailed data on not only drug pharmacokinetics but also carrier fate, biodegradation, immunogenicity, and long term joint safety. However, the penalised cost and complexity of generating such data in large animal models slow progress. Good manufacturing practice (GMP) production of multifunctional nanocarriers (e.g., targeting ligand conjugation, surface modification, stimuli linkers) is non trivial and frequently cited as a bottleneck for translation (Liao et al., 2024). Characterisation standards (sterility, endotoxin, release kinetics, stability under mechanical loading, joint fluid protein binding) remain loosely defined for this niche of IA delivery.

Why IA nanocarriers are harder to scale than conventional pharmaceuticals: unlike small molecules with a limited set of release tests, nanoformulations must control multiple, tightly coupled critical quality attributes (CQAs), including particle size distribution, zeta potential, drug loading/encapsulation efficiency, free (unencapsulated) drug fraction, ligand density and orientation, linker conversion, and long-term colloidal stability under storage and syringeability constraints. Multi-step surface functionalization (e.g., PEGylation and ligand conjugation) can amplify batch-to-batch variability, and some systems are incompatible with terminal sterilization or sterile filtration without altering size distributions and yields. In addition, standardized compendial assays are often insufficient to capture joint-relevant performance, such as stability in synovial fluid, shear- and load-dependent integrity, and trigger-specific release kinetics, necessitating validated in-house analytical methods and more complex CMC packages for regulatory review.

### 7.3 Clinical trial design and implementation

To date, most IA nanodelivery work remains in preclinical phases; early clinical experience is limited. Designing appropriate clinical endpoints for knee OA is non straightforward: structural progression is slow and often detectable only over years, while pain and function outcomes (the primary regulatory endpoints) may not reflect structural modification. Stratification of patients by OA phenotype (inflammatory vs. mechanical vs. metabolic) is rarely integrated in trials, yet nanocarriers targeting specific microenvironment elements would likely perform best in tailored sub populations (Mohammadinejad et al., 2020). Dose, injection schedule, volume, and repeat administration protocols for nanomedicines must be defined in the context of joint mechanics and clearance kinetics unique to human knees. The acceptability of IA injections from patients and the cost effectiveness of advanced nanomedicines compared with standard of care must also be established.

### 7.4 Future perspectives

The future for IA precision targeted nanodelivery in knee OA lies in integrating the fields of personalized medicine, advanced modelling (*in silico*, *in vitro* joint organoids, *ex vivo* human explants), and artificial intelligence assisted design of carriers. Combining multi omics phenotyping (e.g., synovial transcriptomics, cartilage imaging biomarkers) with tailored nanocarriers may enable personalized interventions that match pathophysiology to delivery strategy. Moreover, leveraging artificial intelligence to individualize the optimization of intra-articular nanocarriers—including their size, morphology, surface properties, and release kinetics—based on patient-specific joint biomechanics and tissue characteristics could substantially accelerate clinical translation. This process can be guided by an evidence-driven pathway that first requires rigorous large-animal validation under realistic joint motion and loading conditions, followed by the establishment of standardized protocols for biocompatibility and long-term safety assessment. Early-phase human trials should then characterize pharmacokinetics, retention, and tissue distribution through advanced imaging, after which stratified phase II studies in well-defined osteoarthritis phenotypes can employ combined structural and symptomatic endpoints to enhance sensitivity to treatment effects. Ultimately, cost-effectiveness modeling will be essential to support clinical adoption.

In summary, while precision targeted IA nanodelivery holds transformative potential for knee OA, realizing clinical translation demands concerted efforts spanning design, manufacturing, regulation, and clinical strategy. Coordination among materials scientists, engineers, clinicians, and regulatory agencies will determine whether this promise becomes a reality.

## 8 Conclusion

The integration of precision-targeted nanodelivery systems in the treatment of KOA holds great promise for addressing the multifaceted challenges posed by this debilitating joint disease. Recent advancements in nanotechnology have led to the development of sophisticated drug delivery platforms that can not only enhance the retention and penetration of therapeutics in the knee joint but also offer targeted action to specific tissues, cells, and even molecular pathways implicated in OA progression. These innovations, particularly those incorporating stimuli-responsive release mechanisms and theranostic properties, have the potential to shift the treatment paradigm from merely alleviating symptoms to modifying the disease process itself.

However, significant challenges remain in translating these promising preclinical results into effective clinical applications. Issues related to biocompatibility, long-term safety, and the complex pharmacokinetics of nanomedicines in the knee joint must be addressed before widespread clinical adoption. The variability in OA phenotypes and the heterogeneous nature of disease progression across patient populations further complicates treatment strategies. More work is needed to refine patient stratification techniques and to design nanotherapeutic platforms that can be tailored to individual needs.

The regulatory and manufacturing hurdles associated with complex nanomedicines must also be overcome to facilitate their clinical translation. A clear understanding of the pharmacodynamics, pharmacokinetics, and potential off-target effects of these novel systems will be essential for obtaining regulatory approval. Furthermore, robust clinical trial designs that consider long-term outcomes, including joint function and disease modification, are necessary to ensure the efficacy of these therapies in real-world settings.

In conclusion, while the development of precision-targeted nanodelivery systems for knee OA represents an exciting frontier in the field of regenerative medicine, concerted efforts in both the scientific and regulatory domains are required to overcome the existing challenges. The future of OA therapy will likely see a paradigm shift toward personalized, disease-modifying interventions, and nanomedicine is poised to play a pivotal role in this transformation.
